# Enhanced expression of peroxisome proliferator-activated receptor gamma in epithelial ovarian carcinoma

**DOI:** 10.1038/sj.bjc.6602244

**Published:** 2004-12-07

**Authors:** G Y Zhang, N Ahmed, C Riley, K Oliva, G Barker, M A Quinn, G E Rice

**Affiliations:** 1 Department of Obstetrics and Gynaecology, Qilu Hospital of Shandong University, 107 Wenhuaxi Road, Jinan 250012, PR China; 2 Gynaecological Cancer Research Centre, The Royal Women's Hospital, 132 Grattan Street, Carlton, Victoria 3053, Australia; 3 Department of Obstetrics and Gynaecology, The University of Melbourne, Victoria, Australia

**Keywords:** peroxisome proliferator-activated receptor, ovarian cancer, immunohistochemistry, nuclear and cytoplasmic staining

## Abstract

The peroxisome proliferator-activated receptors (PPARs) belong to a subclass of nuclear hormone receptor that executes important cellular transcriptional functions. Previous studies have demonstrated the expression of PPAR*γ* in several tumours including colon, breast, bladder, prostate, lung and stomach. This study demonstrates the relative expression of PPAR*γ* in normal ovaries and different pathological grades of ovarian tumours of serous, mucinous, endometrioid, clear cell and mixed subtypes. A total of 56 ovarian specimens including 10 normal, eight benign, 10 borderline, seven grade 1, nine grade 2 and 12 grade 3 were analysed using immunohistochemistry. Immunoreactive PPAR*γ* was not expressed in normal ovaries. Out of eight benign and 10 borderline tumours, only one tumour in each group showed weak cytoplasmic PPAR*γ* expression. In contrast, 26 out of 28 carcinomas studied were positive for PPAR*γ* expression with staining confined to cytoplasmic and nuclear regions. An altered staining pattern of PPAR*γ* was observed in high-grade ovarian tumours with PPAR*γ* being mostly localized in the nuclei with little cytoplasmic immunoreactivity. On the other hand, predominant cytoplasmic staining was observed in lower-grade tumours. Significantly increased PPAR*γ* immunoreactivity was observed in malignant ovarian tumours (grade 1, 2 and 3) compared to benign and borderline tumours (*χ*^2^=48.80, *P*<0.001). Western blot analyses showed significant elevation in the expression of immunoreactive PPAR*γ* in grade 3 ovarian tumours compared with that of normal ovaries and benign ovarian tumours (*P*<0.01). These findings suggest an involvement of PPAR*γ* in the onset and development of ovarian carcinoma and provide an insight into the regulation of this molecule in the progression of the disease.

Epithelial ovarian cancer is the leading cause of death from gynaecologic malignancies. As ovarian cancer produces few specific symptoms in the early stage, most women present with advanced stage disease where the prognosis is poor (Jacobs and Menon, 2004). Greater than 90% of epithelial ovarian cancer arises from the transformation of ovarian surface epithelium (Choi and Auersperg, 2003). Hence, comparison between the protein expression profile of normal and transformed ovaries is important to identify and understand the molecules involved in the onset and progression of the disease.

The peroxisome proliferator-activated receptors (PPARs) comprise an important subfamily of the nuclear hormone receptor superfamily. Three isoforms have been identified, PPAR*α*, PPAR*β* and PPAR*γ*. Each exhibits distinct patterns of tissue distribution and ligand specificity (Kersten *et al*, 2000). They share common structural features, which include an amino-terminal modulatory domain, a DNA-binding domain and a carboxyl-terminal ligand-binding domain (Moras and Gronemeyer, 1998). PPAR*α* is present in high levels in the kidney, heart, muscle, liver, brown adipose tissue and gut (Kliewer *et al*, 1994), whereas PPAR*β* is ubiquitously expressed throughout the body (Kliewer *et al*, 1994). PPAR*γ* is highly expressed in adipose tissue, and is also present in other tissues including the muscle, liver, heart, adrenal gland, spleen and placenta (Asami-Miyagishi *et al*, 2004; Feingold *et al*, 2004).

The PPARs are ligand-dependent transcription factors that regulate target gene expression by binding to specific peroxisome proliferator response elements (PPREs) in enhancer sites of target genes (Berger and Moller, 2002). Each receptor binds its PPREs as a heterodimer with a retinoid X receptor. Upon ligand activation, conformational rearrangement of PPAR*γ* expresses transcriptional coactivator binding sites. Recruitment of coactivators involves transcription of genes implicated in the regulation of cell differentiation and activation pathways (Berger and Moller, 2002).

In cancer biology, PPAR*γ* is the most intensively studied PPAR isoform. It is expressed in high levels in different cancer including colon (Bull, 2003), breast (Jiang *et al*, 2003), bladder (Yoshimura *et al*, 2003), prostate (Smith and Kantoff, 2002), head and neck (Jaeckel *et al*, 2001), cervical (Han *et al*, 2003) and endometrial cancer (Tong *et al*, 2000). Recent studies have demonstrated that ligand activation of PPAR*γ* receptor is involved in adipocyte (Seo *et al*, 2004) and tumour cell differentiation (Gauthier *et al*, 2003). In colon cancer, ligand activation of PPAR*γ*-mediated differentiation of certain colon cancer cells results in the upregulation of tumour suppressor genes caveolin 1 and 2 (Burgermeister *et al*, 2003) and repression of cyclin D1 expression (Wang *et al*, 2003). Similarly, *in vitro* studies in prostate cancer cells, which express fairly abundant PPAR*γ*, can result in the differentiation of prostate cancer cells and downregulation of androgen-stimulated PSA production (Hisatake *et al*, 2000). These findings have potentially important functional implication in the context of cancer cell differentiation therapy and multidrug resistance. Recently, Her2 has been shown to regulate PPAR expression (Yang *et al*, 2003). The ligands for PPAR*γ* have been shown to increase the expression of BRCA 1 protein in human breast cancer cells (Pignatelli *et al*, 2003), indicating that PPAR*γ* plays a crucial role in BRCA1 regulatory pathways involved in the pathogenesis of breast and sporadic ovarian cancer.

To our knowledge, a role for PPAR*γ* in ovarian cancer development or function has not been described. In this study, we evaluated immunoreactive PPAR*γ* protein expression in different pathological grades and subtypes of human epithelial ovarian tumours. We show that ovarian tumours express PPAR*γ* and that expression is significantly higher in malignant tumours compared to benign tumours and normal ovaries. We also demonstrate that PPAR*γ* expression pattern alters in high-grade ovarian tumours, implicating an important role for PPAR*γ* in the progression of ovarian malignancy.

## MATERIALS AND METHODS

### Antibody and reagents

Mouse monoclonal and rabbit polyclonal antibodies against PPAR*γ* were obtained from Santa Cruz Biotechnology Inc. (Santa Cruz, CA, USA). Mouse IgG1 was obtained from Sigma (Sigma, St Louis, MO, USA). Immunoperoxidase secondary detection system was obtained from Chemicon International (Temecula, USA). ECL Western blotting detection reagents and analysis system were supplied by Amersham Biosciences (Amersham, UK).

### Immunohistochemistry

The study was approved by the Research and Human Ethics Committee (HEC#02/30) of the Royal Women's Hospital, Melbourne, Australia. Human ovarian tumour tissues were collected at the time of surgical cytoreduction with the informed consent of the patients. Patient information is presented in Table 1 . Normal ovaries for control comparison were collected from patients undergoing surgery as a result of suspicious ultrasound, from prophylactic oophorectomy specimens. The pathology diagnosis and tumour grade was evaluated by two staff pathologists in the Department of Pathology, the Royal Women's Hospital, Melbourne, Australia. The classification of the tumours was performed as part of the clinical diagnosis according to the method described by Silverberg (2000). Surgically removed samples were fixed in 10% formalin and embedded in paraffin. Tissues for Western blot were snap frozen in liquid nitrogen and stored at −80°C until needed.

Paraffin-embedded ovarian tissues were cut at 4 *μ*m thickness and deparaffinised with xylene and rehydrated using graded ethanol. After microwave antigen retrieval in citrate buffer, pH 6.0, the sections were held in Tris buffer solution (TBS, 100 mM, pH 7.6). Endogenous peroxidase activity was inactivated using 3% hydrogen peroxide in methanol and endogenous biotin activity was blocked by a sequence of diluted egg white (5% in distilled water) and skimmed milk powder (5% in distilled water), all for 10 min each. The sections were incubated in mouse monoclonal antibody against PPAR*γ* (1/400 in 1% BSA in TBS) overnight at 4°C. Antibody binding was amplified using biotin and streptavidin HRP for 10 min each and the complex was visualised using diaminobenzidine. The nuclei were lightly stained with Mayer's haematoxylin and the sections were mounted and cover slipped. An isotype IgG_1_ matched diluted was substituted for the antibody as negative control.

Sections were assessed microscopically for positive staining by two experienced observers. For each specimen, the positive staining extent was scored as five grades, namely, 0 (⩽10%), 1 (⩾11–25%), 2 (⩾26–50%), 3 (⩾51–75%), 4(⩾76–90%) and 5 (⩾91–100%) (Armes *et al*, 1999). The intensity was classified into four grades: no staining, negative (−); pale brown, weak (+); brown, moderate (++) and dark brown, strong (+++). Parallel paraffin-embedded sections were stained with haematoxylin and eosin to confirm the pathologic diagnosis simultaneously.

### Western blot

Preparation of ovarian tissue homogenate was performed as described previously (Ahmed *et al*, 2004). Each frozen ovarian specimen (100 mg) was cut into several small pieces about 3 mm in size. Then, the specimen was homogenized in Tris-HCl buffer (10 mM Tris, 150 mM NaCl, 2 mM EDTA, 2 mM dithiothreitol, 1 mM orthovanadate, 1 mM phenylmethylsulphonyl fluoride, 5 *μ*g ml^−1^ aprotonin, pH 7.0) by repeated uniform strokes (approximately 6). The samples were centrifuged at 10 000 **g** for 20 min. The supernatant was collected and relative protein concentration was determined using Bio-Rad Protein Assay Reagent following the manufacturer's instruction. Ovarian homogenate containing equal amounts (10 *μ*g) of protein were separated by electrophoresis on 10% sodium dodecyl sulphate (SDS)–polyacrylamide gels under nonreducing condition and transferred to nitrocellulose membranes. The membranes were probed with rabbit polyclonal anti-PPAR*γ* (diluted 1 : 400 in 3% skim milk in TBST) followed by peroxidase-labelled donkey anti-rabbit secondary antibody (1 : 2500) and visualised by the ECL (Amersham, UK) detection system according to the manufacturer's instruction.

### Statistical analysis

The extent and intensity of immunohistochemical staining between benign, borderline and high-grade ovarian tumours was determined by *χ*^2^ test. The association between the optical density (OD) of PPAR*γ* bands determined by Western blotting in benign tumours compared to that of high-grade ovarian tumours was assessed by Student's *t*-test.

## RESULTS

### Immunohistochemical staining of PPAR*γ* in ovarian tumour tissues

**Figure 1:**
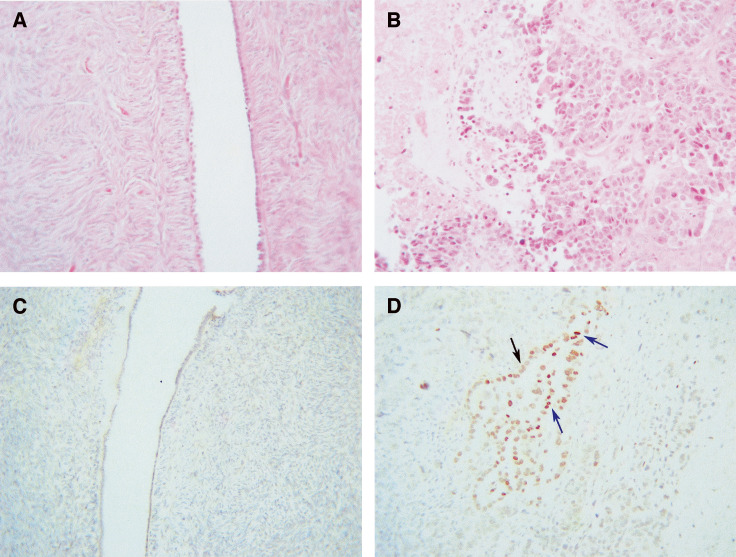
(**A** and **B**) Haematoxylin and eosin staining of (**A**) normal ovary, (**B**) grade 3 serous tumour. PPAR*γ* staining of the same (**C**) normal ovary (**D**) and grade 3 serous ovary. Blue arrows indicate nuclear PPAR*γ* staining, while black indicates cytoplasmic staining.

**Figure 2:**
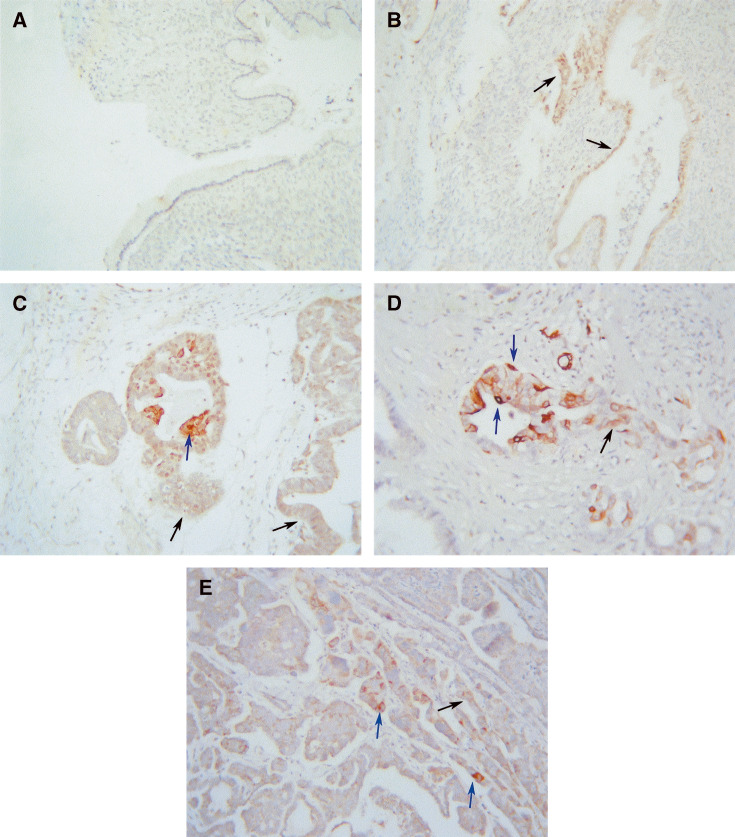
PPAR*γ* staining of (**A**) benign mucinous tumour, (**B**) grade 1 mucinous tumour, (**C**) grade 2 endometrioid tumour, (**D**) grade 3 endometrioid tumours and (**E**) grade 3 serous tumour. Arrows indicate nuclear (blue) and cytoplasmic (black) PPAR*γ* staining.

Immunohistochemical expression of PPAR*γ* in epithelial ovarian tumours is described in Table 2 . No immunoreactivity of PPAR*γ* was observed in normal ovarian tissues (Figure 1C). Among eight benign and 10 borderline ovarian tumours, weak PPAR*γ* expression was present in only one tumour in each group (Figure 2A). Seven cases of grade 1 ovarian tumours were studied. Four of these showed weak staining and moderate staining was observed in three cases. Both cytoplasmic and nuclear staining was observed (Figure 2B), the distribution being approximately 60% cytoplasmic and 40% nuclear.

Eight out of nine grade 2 tumours studied were positive for PPAR*γ* and the expression varied from a score of 1 to 3 with increased demonstration of nuclear staining (Figure 2C). Nuclear staining of infiltrating macrophages was observed in one case. In all, 12 grade 3 ovarian tumours were studied and 11 of these were positive. The intensity of PPAR*γ* staining varied but was predominantly judged to be moderate (++) (Figures 1D and 2D, E). In grade 2 and 3 tumours, staining was predominantly nuclear. Cytoplasmic staining in higher grades (grades 2 and 3) represented approximately 20% of total staining.

Overall, the immunoreactive PPAR*γ* was present in all grades of ovarian tumours. The extent of overall staining was significantly higher in malignant ovarian tumours (grades 1, 2 and 3) compared with benign and borderline tumours (*χ*^2^=48.80, *P*<0.001) (Table 2). PPAR*γ* staining in ovarian tumours was mainly localised to the cytoplasm or nuclei of tumour cells. Nuclear staining increased significantly with the grade of the tumours (80% in grade 3 compared to 40% in grade 1). Tumour stroma or endothelial cells lining the blood vessels were negative for PPAR*γ* immunoreactivity. The intensity of PPAR*γ* staining was also significantly higher in malignant tumours compared with benign and borderline tumours (*χ*^2^=43.93, *P*<0.001) (Table 2). No statistical difference in the extent or intensity of staining was observed between the different grades of tumours (grades 1, 2 and 3) (*χ*^2^=4.29, *P*=0.6363; *χ*^2^=1.68, *P*=0.79) (Table 2).

### Expression of PPAR*γ* in ovarian tumour tissues using Western blot analysis

**Figure 3:**
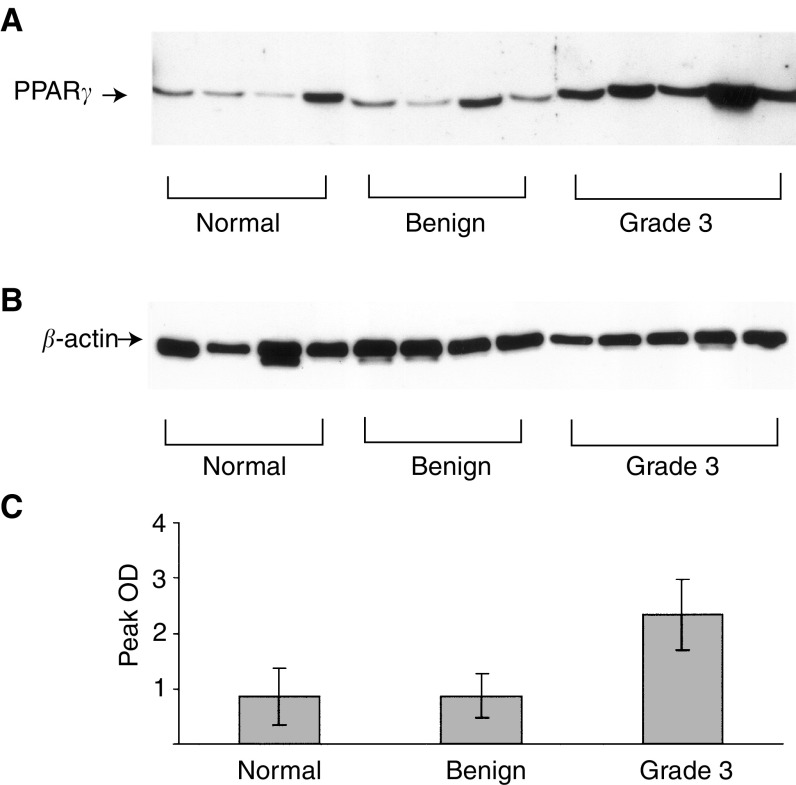
Western blot analyses of PPAR*γ* expression in normal ovary, benign and grade 3 ovarian tumours. (**A**) Western blot was carried out as described in the Material and Methods section. A total of 10 *μ*g of protein was loaded in a total volume of 20 *μ*l in each lane. The results are representative of one experiment repeated three times. (**B**) *β*-actin staining of the same samples loaded in same concentration to ensure equal protein loading. (**C**) Quantification of PPAR*γ* expression was performed by densitometry and expressed as mean peak OD±s.e.m. of the number of samples described in each group.

The expression of PPAR*γ* in human ovarian tumour tissues was also evaluated by Western blot analyses (Figure 3). The expression of PPAR*γ* in ovarian tumour tissues was significantly higher than in normal ovaries and benign ovarian tumours (*P*<0.01) (Figure 3A). However, no difference in the expression of PPAR*γ* in normal ovaries and benign ovarian tumours was demonstrated (*P*>0.05) (Figure 3C).

## DISCUSSION

The sustainability of a malignant tumour requires multiple cellular events by which the cancer cells acquire growth factor independence, escape cellular apoptosis mechanisms, create a self-sustaining environment and escape the neighbouring barriers by migrating and colonising to a distant site (Hanahan and Weinberg, 2000). These events require the expression/overexpression and activation of molecules not generally requisite for normal cellular functions. The initial indication that the PPARs are involved in the aetiology of cancer was the isolation of PPAR*α* as the mediator of the tumour-promoting effect of peroxisome proliferators, compounds that cause heptocellular carcinoma in rodents (Corton *et al*, 2000). PPAR*α* has been shown to be expressed in colon tumours and overexpressed in breast and prostate tumours (Collett *et al*, 2000; Roberts-Thomson and Snyderwine, 2000). PPAR*β* levels are also elevated in colon and head and neck cancer (Gupta *et al*, 2000; Jaeckel *et al*, 2001) and the absence of PPAR*β* reduces tumour growth in colon cancer (Park *et al*, 2001). Although PPAR*γ* is expressed at low levels in normal colonic and breast ductal epithelium, it is significantly increased in breast and prostate carcinoma. The expression of this receptor has not been reported in ovarian carcinoma but has been shown in normal ovaries (Lambe and Tugwood, 1996). In this study, we report the expression of PPAR*γ* in different pathological grades and subtypes of ovarian carcinoma and discuss its possible function with the progression of the disease.

We report for the first time that ovarian tumours express PPAR*γ*. Weak to moderate expression of PPAR*γ* by immunohistochemistry was observed in almost all ovarian tumours studied. As shown in Table 2 and Figure 1, immunohistochemical staining showed no PPAR*γ* expression in normal ovarian tissues. Out of eight benign and 10 borderline tumours, only one in each group stained positive for PPAR*γ* expression. Weak to moderate staining was observed in grade 1 ovarian tumours (Figure 2B), and the staining was localised to both cytoplasmic and nuclear areas of the cells. Compared to benign and borderline tumours, the extent of staining was, however, significantly increased in grade 3 ovarian tumours, with immunoreactivity for PPAR*γ* being mostly present in the nuclear region (Figures 1 and 2). Western blotting analyses demonstrated significant enhancement in the expression of PPAR*γ* in grade 3 ovarian tumours compared to benign ovarian tumours and normal ovaries (Figure 3). The basal expression of PPAR*γ* in normal ovarian tissues and benign ovarian tumours may have been attributed by the increased immunosensitivity of Western blotting technique compared to immunohistochemistry.

The results from our study are consistent with those of other studies performed in other cancers. Very weak immunohistochemical staining of PPAR*γ* was shown in benign prostatic hyperplasia and normal prostate tissues, whereas significant enhancement in the expression of immunoreactive PPAR*γ* was observed in malignant prostate tissues (Park *et al*, 2001). PPAR*γ* expression was shown to be higher in high-grade bladder cancer compared to low-grade cancer (Yoshimura *et al*, 2003). Irrespective of the differentiation status of the tumour, strong expression of immunoreactive PPAR*γ* was observed in surgically resected human gastric cancer tissues (Sato *et al*, 2000).

In contrast, in some cases of cancer, the expression of PPAR*γ* decreases with the histological grade of the tumour. In full-term normal placenta, PPAR*γ* is strongly expressed in the nuclei of the syncytiotrophoblast, extravillous cytotrophoblast of cell islands and cell columns, whereas in choriocarcinoma, only a few trophoblastic cells show weak staining for PPAR*γ* (Capparuccia *et al*, 2002). Well-differentiated lung adenocarcinomas present increased frequency for PPAR*γ* expression compared with moderately and poorly differentiated ones (Theocharis *et al*, 2002). The expression of PPAR*γ* protein is decreased in oesophageal cancer tissues compared with normal oesophageal squamous epithelium (Terashita *et al*, 2002). Hence, considering the diversity of human cancer, the expression of PPAR*γ* is possibly dependent on tissue specificity and/or the mutational events (as in the case of colon cancer) (Ikezoe *et al*, 2001) that are requisite for cancer development.

The growth inhibitory and differentiation roles of PPAR*γ* have been shown in several cancers (Demetri *et al*, 1999). The immunohistochemical expression of PPAR*γ* during the progression of ovarian cancer can be related to the growth-promoting role of PPAR*γ* previously shown in certain cancer (Mueller *et al*, 2000). In the case of thyroid follicular cancer, a chromosomal translocation and fusion of PAX8 gene with PPAR*γ* results in a malignant phenotype, suggesting a link of PPAR*γ* to cancer growth (Dwight *et al*, 2003). In the Min mouse model of APC deficiency, ligands for PPAR*γ* can increase colon tumour growth (Lefebvre *et al*, 1998). In another study, loss of PPAR*γ* was shown not to affect mammary development and propensity for tumour formation but resulted in reduced fertility (Cui *et al*, 2002). Overall, ligand activation of PPAR*γ* in tumour models can result in diversified functional outcome (Leung *et al*, 2004) and a better understanding of the exact role of PPAR*γ* in cancer needs to be determined. In particular, the mechanism of differentiation of cancers is incompletely understood and an insight into this process would undoubtedly lead to new therapeutic targets.

The anti-inflammatory response of PPAR*γ* in association with NF-*κ*B has recently been identified (Kelly *et al*, 2004). In addition, PPAR*γ* ligands have been shown to inhibit transcriptional activation of COX-2 in human epithelial cells (Badawi *et al*, 2004). In certain cancer cells, ligand activation of PPAR*γ* results in the inhibition of the release of inflammatory cytokines by cancer cells (Leung *et al*, 2004). These results may help to explain the shuttling of PPAR*γ* expression from the cytoplasm to nucleus with the progression of ovarian carcinoma. As ovarian cancer progresses, tumour cells are more likely to be exposed to inflammatory cytokines secreted by cancer cells themselves and infiltrating leucocytes present in the peritoneum. Whether this increased exposure of ovarian tumour cells to the inflammatory cytokines result in the activation of PPAR*γ* and subsequent cytoplasmic translocation to the nucleus is yet to be determined.

Taken together, our results indicate that PPAR*γ* may play a role in the onset and progression of ovarian cancer. Further research need to investigate the prognostic significance of PPAR*γ* expression in ovarian carcinoma and whether therapeutic administration of ligands for this receptor may have clinical potential for the treatment of the disease.

## Figures and Tables

**Table 1:** Description of ovarian cancer patients participating in the study

**Case no.**	**Histological grade**	**Clinical stage**	**Tumour subtype**	**CA125 (U ml^−1^)**
6	1	1a	Endometrioid	773
64	1	1b	Endometrioid	786
83	1	1c	Endometrioid	126
106	1	3c	Mixed	93
164	1	1b	Endometrioid	636
190	1	1a	Mucinous	25
198	1	2c	Endometrioid	55
32	2	1a	Endometrioid	302
35	2	3c	Serous	1200
46	2	4	Serous	3457
49	2	3c	Serous	900
110	2	3c	Serous	1719
121	2	3c	Serous	3471
123	2	3b	Mixed	106
129	2	3c	Mixed	3823
161	2	1c	Mixed	698
2	3	3c	Serous	647
3	3	3c	Serous	513
44	3	2b	Endometrioid	165
85	3	1a	Serous	147
88	3	3c	Serous	883
90	3	3c	Endometrioid	659
96	3	3c	Serous	137
108	3	3c	Serous	61
143	3	4	Clear cell	507
146	3	4	Serous	2463
153	3	4	Serous	985
158	3	3c	Serous	287

**Table 2:** Extent and intensity of PPAR*γ* expression in normal ovaries and tumour tissues

**Histology**	**Total number of tissues**	**Extent of staining (number of tissues)**	**Intensity of staining (number of tissues)**
Normal	10	0 (10)	− (10)
Benign	8	0 (7), 1 (1)	− (7), + (1)
Borderline	10	0 (9), 1 (1)	− (9), + (1)
Grade 1	7	0 (0), 1 (6), 2 (0), 3 (1)	− (0), + (3), ++ (4)
Grade 2	9	0 (1), 1 (5), 2 (1), 3 (2)	− (1), + (5), ++ (3)
Grade 3	12	0 (1), 1 (5), 2 (1), 3 (5)	− (1), + (7), ++ (4)
			
Total	56	56	56

PPAR=peroxisome proliferator-activated receptor.

Extent of PPAR*γ* expression was scored as 0 (⩽10%), 1 (⩾11–25%), 2 (⩾26–50%), 3 (⩾51–75%), 4(⩾76–90%) and 5 (⩾91–100%) immunoreactivity. Values within parentheses indicate number of tissues in each category. The extent of staining was significantly different in grade 1, 2 and 3 tumours compared to benign and borderline tumours (*χ*^2^=48.80; *P*<0.001). The intensity of staining was scored as negative (−), weak (+), moderate (++) and strong (+++) staining. The values within parentheses indicate the number of tissues in each category. The intensity of staining was significantly different in grade 1, 2 and 3 tumours compared to benign and borderline tumours (*χ*^2^=43.93; *P*<0.001).
